# The Effect of Dietary Supplementation with Inorganic or Organic Selenium on the Nutritional Quality and Shelf Life of Goose Meat and Liver

**DOI:** 10.3390/ani11020261

**Published:** 2021-01-21

**Authors:** Zabihollah Nemati, Kazem Alirezalu, Maghsoud Besharati, Benjamin W. B. Holman, Mohammadreza Hajipour, Benjamin M. Bohrer

**Affiliations:** 1 Department of Animal Science, Ahar Faculty of Agriculture and Natural Resources, University of Tabriz, Tabriz 5166616471, Iran; m_besharati@hotmail.com (M.B.); mrezahajipour@yahoo.com (M.H.); 2 Department of Food Science and Technology, Ahar Faculty of Agriculture and Natural Resources, University of Tabriz, Tabriz 5166616471, Iran; kazem.alirezalu@yahoo.com; 3 Centre for Red Meat and Sheep Development, NSW Department of Primary Industries, Cowra, NSW 2794, Australia; benjamin.holman@dpi.nsw.gov.au; 4 Department of Animal Sciences, The Ohio State University, Columbus, OH 43210, USA

**Keywords:** dietary selenium, geese, goose meat, goose quality, goose liver, inorganic selenium, organic selenium, selenium-enriched yeast

## Abstract

**Simple Summary:**

Geese have a unique ability among aquatic poultry species to efficiently utilize high-fiber feedstuffs, however research investigating concentrate feeding strategies in the farm setting is limited. This experiment investigated the effect of dietary supplementation with inorganic or organic selenium on nutritional quality and shelf life of goose meat and liver samples. Differences between geese supplemented with I-Se and O-Se were detected for several parameters, yet these differences were less tangible than those between geese not supplemented with additional selenium (CON) and geese supplemented with additional selenium (I-Se and O-Se). Overall, it was concluded that supplementation with additional dietary selenium in both the inorganic and organic forms improved nutritional quality and shelf life of goose meat and liver samples.

**Abstract:**

Ninety-six male goslings were allocated and assigned to treatment using a completely randomized design. Dietary treatments included a basal diet consisting of corn, wheat, and soybean meal with either no additional selenium (CON), 0.3 mg/kg of inorganic selenium (I-Se; sodium selenite), or 0.3 mg/kg of organic selenium (O-Se; selenium-enriched yeast). After a 56-day feeding period, geese were slaughtered on a common ending day and two geese per pen (*n* = 24) were used for the analyses conducted in this study. Meat (equal portions of the breast and thigh meat) and liver were collected and evaluated for proximate composition, fatty acid profile, pH, phenolic content, thiobarbituric acid reactive substances (TBARS), and total volatile basic nitrogen (TVB-N) over a 9-day storage period at 4 °C. The meat and liver samples from geese supplemented I-Se or O-Se had greater (*p* < 0.01) lipid content compared with geese not supplemented with additional selenium. At the conclusion of the 9-day storage period, meat and liver samples from geese supplemented I-Se or O-Se had lower (*p* < 0.05) pH values, greater (*p* < 0.05) phenolic content, lower (*p* < 0.05) TBARS values, and lower (*p* < 0.05) TVB-N compared with geese not supplemented with additional selenium (CON).

## 1. Introduction

The reasons for the domestication of geese into sustainable agricultural systems include their ability to efficiently utilize high-fiber feedstuffs, their ease of production due to generally docile behavior, and their rapid growth rates–which are among the fastest growth rates in domesticated avian species [1,2]. The role of dietary selenium has been well documented in domesticated poultry species [3,4,5]. The National Research Council (NRC) [3] does not list dietary selenium requirements for geese, but the dietary selenium requirement for chickens is listed as 0.15 mg/kg for 0- to 6-week-old birds and 0.10 mg/kg for birds older than 6 weeks. The maximum level of supplemental selenium that can be fed to poultry is regulated by government agencies in the United States, Canada, Europe, and most other countries. The maximum level of supplemental inorganic selenium (sodium selenite) that can be fed to poultry in most countries is 0.3 mg/kg [6]. The maximum level of supplemental organic selenium (selenium-enriched yeast or selenomethionine) that can be fed to poultry is 0.3 mg/kg in North America [7] and 0.5 mg/kg in Europe [8]. The primary difference between inorganic and organic trace minerals is summarized by differing levels of absorption and bioavailability; where inorganic sources typically have lower absorption and bioavailability compared with organic sources [9,10,11]. Inorganic-sourced selenium is released and may re-combine with other nutrients in the intestine making insoluble complexes that are excreted, which reduces the absorption of selenium in the small intestine whereas organic-sourced selenium is actively absorbed, which allows for peptide and/or amino acid uptake mechanisms in the intestine to be utilized [12]. Key research initiatives in recent decades for several different domesticated livestock species include investigation into the appropriate level and source (inorganic versus organic) for selenium supplementation.

Selenium is closely associated with vitamin E and other antioxidants found in feed ingredients, and has an integral role in reproductive performance, animal health, and animal growth and development [3,13]. Selenium is an important constituent of the enzyme glutathione peroxidase, which acts to destroy peroxides thereby protecting cells and membranes against oxidative damage caused by stress or other unfavorable situations during production [14,15,16]. Vitamin E and selenium tend to enhance the effect of one another since vitamin E works to prevent the formation of peroxides, while glutathione peroxide, as aforementioned, acts to destroy peroxides [17,18,19]. Lipid oxidation is a challenge for the poultry industry as it contributes to the degradation of unsaturated fat in meat and egg products during storage [20,21]. The development of several different rancid flavors and aromas accompany the degradation of unsaturated fats [22,23,24]. Feeding antioxidants to poultry is a simple way to improve their oxidative status and increase its retention in meat products [25].

There have been several recent studies with promising results when broiler chickens were fed elevated levels of selenium, and especially when organic sources of selenium were used [26,27,28,29,30,31]. Choct et al. [26] reported increased levels of dietary selenium markedly improved feed conversion rate and meat yield in broiler chickens. Furthermore, the authors [26] reported that organic selenium (selenium-enriched yeast) was superior to inorganic selenium (sodium selenite) due to greater absorption levels of selenium in serum and tissues. Yoon et al. [27] and Wang et al. [28] reported that organic selenium (selenomethionine) was more effective (greater absorption in serum and tissues) than inorganic selenium (sodium selenite), thus enhancing the antioxidant status of meat tissue and improving meat quality. Furthermore, Yuan et al. [29] studied selenium supplementation in broiler breeders and chickens and reported that organic selenium (selenium-enriched yeast and selenomethionine) increased the activity of thyroidoxin reductase in the liver and kidney when compared with inorganic selenium (sodium selenite). 

There have been a few studies evaluating selenium supplementation in geese. Lukaszewicz et al. [32] reported that geese supplemented with organic selenium and vitamin E at levels of 0.3 mg/kg and 100 mg/kg, respectively, had improved growth rate but allometric muscle growth was unaffected. Baowei et al. [33] reported geese supplemented with organic selenium (selenium-enriched yeast) had greater selenium content in their liver, kidney, pancreas, and muscle which resulted in improved antioxidant capacity of the meat products (greater glutathione peroxidase activity). However, existing research on selenium supplementation of geese does not extend to important aspects related to applied meat quality.

In summary, existing studies demonstrate the expanding knowledge and understanding as to the effects of selenium supplementation on poultry growth, development, and meat quality–yet limitations still exist in reference to poultry species beyond that of chickens. Therefore, the objective of this study was to determine the effect of dietary supplementation with inorganic or organic selenium on nutritional quality and shelf life of goose meat and liver.

## 2. Materials and Methods

All procedures were approved by the Research Bioethics Committee of the University of Tabriz.

### 2.1. Experimental Design and Treatment

A total of 96 one-day old male goslings with the initial body weight of 92.5 ± 2.5 g were selected for inclusion in the 56-day feeding experiment. Specifically, this study used Azerbaijan native goslings obtained from the Goose Research Station of Malekan (Malekan, Iran). The Azerbaijani variety of local Iranian geese is supported by the Malekan Goose Research Station under the supervision of the Ministry of Jihad Agriculture. This variety of geese can be characterized by high levels of live performance and high levels of meat yield. In fact, geese from this research flock have been used in another recent study [19], please reference this study for typical expectations for live production and carcass composition measures for native Azerbaijan geese. Goslings were randomly assigned to 1 of 3 dietary treatment groups. A total of 12 floor pens were used (4 pens per treatment), and each pen housed 8 goslings. Feed and water were provided ad libitum throughout the experiment. Table 1 presents the chemical composition of the basal diet, which was composed of corn, wheat, and soybean meal formulated according to the nutritional requirements of geese as referenced in the NRC [3].

Dietary treatments used in this study were: (1) the basal diet with no additional selenium (CON); (2) the basal diet supplemented with 0.3 mg/kg of inorganic selenium (I-Se; sodium selenite, Merck Serona GmbH, Darmstadt, Germany); and (3) the basal diet supplemented with 0.3 mg/kg of organic selenium (O-Se; selenium-enriched yeast, Selplex, Alltech, Nicholasville, KY, USA).

On the last day of the experiment, two geese from each pen (*n* = 24 geese; 8 geese per treatment) were randomly selected for slaughter. The slaughtering process followed a 12-h lairage period and was conducted with the same procedures previously described by Cui et al. [34]. Following a 2-min exsanguination period, carcasses were scalded in 60 °C water for 2 min prior to mechanical feather plucking, evisceration, and tissue sample collection. Both breasts (IMPS #P4015), both thighs (IMPS #P4033), and the liver (IMPS #P4045) were collected for further sampling [35]. Samples were trimmed of subcutaneous fat and sectioned into 2 cm × 2 cm × 2 cm which were immediately packaged in aerobic polypropylene bags and stored at 4 °C. A composite meat sample consisting of equal weights of the breast and thigh samples was generated during this time. Macronutrient composition, pH, total phenolic content, lipid oxidation (thiobarbituric reactive substances; TBARS), and total volatile base-nitrogen (TVB-N) of meat (equal portions of the breast and thigh meat) and liver samples were analyzed at 1-day, 3-day, 6-day, and 9-day intervals during the post-mortem storage period in which the samples were stored in the aerobic polypropylene bags at 4 °C. Meat (equal portions of the breast and thigh meat) samples were immediately collected and frozen for the determination of fatty acid profile.

### 2.2. Composition (Macronutrients and Fatty Acid Profile)

Moisture (oven drying at 102–105 °C), lipid (Soxhlet extraction), protein (Kjeldahl apparatus), and ash (muffle furnace at 500–550 °C) of meat samples and liver samples were analyzed in accordance with the Association of Official Analytical Chemists (AOAC) methodology [36].

Duplicate meat samples were minced, and their lipid content was extracted in accordance with Folch et al. [37]. The fatty acid composition of these lipid extractions was then determined with gas chromatography. The separation of fatty acid methyl esters (FAMEs) was carried out with an Agilent capillary column (30 m × 0.25 mm I.D., CPS Analitica, Milan, Italy) as reported by Pintado et al. [38]. Individual FAMEs were identified based on the retention time of tridecanoic acid (C13:0) methyl ester which was added before extraction as an internal standard. The FAMEs were calculated by comparison of their retention times and peak areas were reported as mg fatty acid/100 g meat sample as previously reported by Alirezalu et al. [39].

### 2.3. pH

pH of meat samples and liver samples was measured according to the methodology described by Torrescano et al. [40]. This involved the homogenization of samples with distilled water (1:10 dilution) using a homogenizer (IKA, T50 Ultra-Turrax, Werke, Staufen, Germany) set at 12,000 revolutions per minute for a 2-min period. pH of the sample dilutions was determined using a calibrated pH meter (Hanna pH model 211, Hanna Instruments, Woonsocket, Rhode Island, USA) with using automatic temperature compensation.

### 2.4. Total Phenolic Content

Determination of total phenolic content of meat samples and liver samples were performed with the Folin Ciocalcio (F-C) method. First, five-gram samples were minced and combined with 10 mL of boiled distilled water (100 °C) where they were held for a 20-min incubation period. These samples were then cooled and filtered using Whatman No. 1 filter paper into a sterilized test tube. Next, 250-mL of F-C reagent and 5 mL of saturated sodium carbonate solution was added, and the solution was vortexed. Samples were then held at room temperature (approximately 22 °C) in the dark for a 1-h incubation period. The sample absorbance at 700 nm wavelength was recorded using a benchtop spectrophotometer (Hitachi, Ltd., Tokyo, Japan) and this outcome was compared against the calibration curve. Gallic acid was used in the range of 0.00 to 0.03 mg/mL, and the line regression was determined (0.0689, R^2^ = 0.9702 Y = 0.9575x). The total phenolic content was expressed as mg/100 g of dry matter as in previous studies [19,41].

### 2.5. Lipid Oxidation

Lipid oxidation was quantified using thiobarbituric acid reactive substances (TBARS) methodology to determine the amount of malondialdehyde (MDA). This analysis used 3 g of minced samples mixed with 5 mL of 20% TCA and 4 mL of distilled water, using an ultraturax homogenizer for 30 s. Samples were then centrifuged at 112× *g* for 20 min (Universal model 320 R, Andreas Hettich GmbH & Co., Tuttlingen, Germany). The supernatant was then isolated and filtered (Platinum DV-24N-250) with Whatman No. 1 filter paper. Within individual test tubes, 2 mL of the filtered supernatant was combined with 2 mL of 2-Thiobarbituric acid (2.0 M) and this was incubated in a 95 °C water bath for 20 min. Samples were allowed to cool before their TBARS content was determined using a benchtop spectrophotometer (Jenway model 6405, Cole-Parmer, Staffordshire, UK) set to measure the absorbance at 532 nm. The results were expressed as mg of malondialdehyde (MDA) per kilograms of product.

### 2.6. Total Volatile Nitrogen (TVB-N)

The Kjeldahl method was used to calculate TVB-N. The TVB-N content of meat samples and liver samples was performed according to methods previously described by Harold et al. [42]. Briefly, 10 g of minced sample was mixed with 250 mL of distilled water and then 2 g of magnesium oxide (Sigma-Aldrich, Darmstadt, Germany) was mixed in a 500 mL Kjeldahl flask. The distilled ammonia borate (300 mL) was titrated using hydrochloric acid solution (0.01 N) in the presence of a mixing indicator (bromocrysol green/methyl red). Results were calculated as mg nitrogen per 100 g of sample.

### 2.7. Statistical Analysis

Data were analyzed with the MIXED procedure of SAS (SAS Inst. Inc., Cary, NC, USA). Macronutrient composition, pH, total phenolic content, lipid oxidation (TBARS), and TVB-N were analyzed as a completely randomized design with treatment and storage day as fixed effects and replication as a random effect. Fatty acid profile was also analyzed as a completely randomized block design, apart from the day effect. Comparison of least squares means was conducted at the statistical level of *p* < 0.05 using a Tukey-Kramer adjustment for multiple comparisons.

## 3. Results and Discussion

### 3.1. Macronutrient Composition of Meat Samples

It was hypothesized that feeding geese supplemental selenium (and particularly organic selenium) would increase the lipid content in the breast and thigh meat samples, while having limited to no effects on the composition of other macronutrients (i.e., moisture, protein, and ash). This hypothesis was founded based on previous research findings on broiler chickens and turkeys. Ševčíková et al. [43] reported greater intramuscular fat content (10.93 g/kg vs. 9.78 g/kg; difference of 1.15 g/kg) and greater abdominal fat (15.0 g vs. 13.8 g; difference of 1.2 g) for the breast muscle in broiler chickens supplemented with 0.3 mg/kg of selenium-enriched alga chlorella compared with broiler chickens not supplemented with selenium, while moisture and protein content of the breast muscle were not significantly affected. Bou et al. [44] reported greater crude fat content (10.9% vs. 10.6%; difference of 0.3 percentage units) for the breast muscle in broiler chickens supplemented with 0.2 mg/kg of selenium-enriched yeast compared with broiler chickens not supplemented with selenium. Mikulski et al. [45] reported greater intramuscular lipid content (0.71% vs. 0.54%; difference of 0.17 percentage units) for the breast muscle of turkeys supplemented with 0.3 mg/kg of organic selenium (selenium-enriched yeast) compared with turkeys supplemented with 0.3 mg/kg of inorganic selenium (selenium selenite). Metabolically, the proposed increase in lipid content associated with supplementation of selenium is not well understood. Glutathione peroxidase and other enzymatic systems that influence antioxidant activity have not been suggested to affect carcass composition or carcass yields [4].

The effect of selenium supplementation on proximate composition of goose meat samples was presented in Table 2.

Moisture, protein, lipid, and ash were significantly affected by treatment on each day of evaluation. There were not consistent trends for moisture of the meat samples during the storage period. Moisture of the meat samples was greater (*p* < 0.01) for geese supplemented I-Se compared with geese fed CON or geese supplemented O-Se on day-1 of storage. Moisture of the meat samples was greater (*p* < 0.01) for geese fed CON compared with geese supplemented I-Se or O-Se on day-3 of storage. Moisture of the meat samples was greater (*p* < 0.01) for geese fed CON compared with geese supplemented I-Se or O-Se on day-6 of storage. Moisture of the meat samples was greater (*p* < 0.01) in geese supplemented O-Se compared with geese fed CON on day-9 of storage. Protein of the meat samples was greater (*p* < 0.01) for geese supplemented O-Se compared with geese fed CON or geese supplemented I-Se on all storage days. The magnitude of difference ranged from 0.65 percentage units to 1.21 percentage units. Selenium supplementation resulted in substantially greater lipid content in the meat samples. Lipid in the meat samples was greater (*p* < 0.01) for geese supplemented I-Se and O-Se compared with geese fed CON on day-1 of storage, and the magnitude of difference was 5.63 percentage units and 4.93 percentage units, respectively. On day-1 of storage, there was not a significant difference for lipid in the meat samples between geese supplemented I-Se and O-Se. For the remaining days of the storage period (day-3, day-6, and day-9), a similar magnitude of difference for lipid in the meat samples was observed between the treatments; however, a significant difference was also detected between geese supplemented I-Se and O-Se where geese supplemented I-Se had greater (*p* < 0.01) lipid content in the meat samples compared with geese supplemented O-Se. There were not consistent trends for ash content of the meat samples during the storage period. Ash content of the meat samples was greater (*p* < 0.01) for geese fed CON compared with geese supplemented I-Se or O-Se on day-1, day-3, and day-9 of storage, and ash content of the meat samples was greater (*p* < 0.01) for geese supplemented O-Se compared with geese fed CON or geese supplemented I-Se on day-6 of storage.

Storage day significantly affected moisture content of the meat samples, but protein, lipid, and ash of the meat samples were unaffected by storage day. Moisture of the meat samples decreased (*p* < 0.05) over the storage period for each treatment. This was expected provided the packaging used in this study (aerobic polypropylene bags and a storage temperature of 4 °C) and has been previously reported by other studies using similar packaging techniques [46].

Overall, it was surprising that geese supplemented I-Se and O-Se had such large increases in lipid content in the meat samples when compared with CON samples. Selenium supplementation in other poultry species has not generated such profound impacts on lipid composition [4,47], and thus greater research efforts are warranted.

### 3.2. Macronutrient Composition of Liver Samples

Selenium supplementation in poultry is known to influence multiple metabolic pathways producing many compounds that are ultimately synthesized or metabolized in the liver [48]. The extent in which the activity caused by upregulation of metabolic pathway signaling in the liver influences liver composition is relatively unknown for more commonly researched poultry species like chicken and turkeys, let alone geese.

The effect of selenium supplementation on proximate composition of goose liver tissue was presented in Table 3. Moisture, protein, lipid, and ash of liver tissue were significantly affected by treatment on each day of evaluation. Moisture of liver tissue was greater (*p* < 0.01) in geese fed CON and geese supplemented O-Se compared with geese supplemented I-Se on each of the storage days. Protein of liver tissue was greater (*p* < 0.01) in geese supplemented O-Se compared with geese fed CON or geese supplemented I-Se on each of the storage days and the magnitude of difference ranged from 2.11 percentage units to 2.49 percentage units. Lipid content of liver tissue was greater (*p* < 0.01) in geese supplemented I-Se and O-Se compared with geese fed CON on each of the storage days and the magnitude of difference ranged from 1.97 percentage units to 2.22 percentage units. Ash content of liver tissue was greater (*p* < 0.01) in geese fed CON compared with geese supplemented I-Se and O-Se. Storage day significantly affected moisture content and ash content of liver tissue, but protein and lipid of liver tissue were unaffected by storage day. Moisture of liver tissue decreased (*p* < 0.05) over the storage period for each treatment, while ash content of the liver tissue decreased (*p* < 0.05) over the storage period for geese supplemented I-Se and O-Se treatments.

Overall, geese supplemented with both sources of selenium (I-Se and O-Se) had greater lipid content in the liver compared with geese not supplemented with selenium (CON), and geese supplemented O-Se had greater protein in the liver compared with geese fed CON and geese supplemented I-Se. These are noteworthy findings as liver composition is an important consideration for acceptable quality of *foie gras* and other further processed liver products. It should be noted that the geese in this study were not reared in a traditional *foie gras* production system (i.e., force-feeding), and the livers were not fully processed into *foie gras*. To this point, the amount of lipid found in the liver in this study (6.21% to 8.50% lipid) was well below industry standard for “raw goose fatty liver” from geese reared in traditional *foie gras* production systems (44.3% to 59.1% lipid) [49,50]. Greater investigation of the role of selenium in these two capacities is warranted.

### 3.3. Fatty Acid Profile

The fatty acid profile of meat samples is reported in Table 4. Many of the individual fatty acids evaluated in this study were affected by treatment. To summarize, there were greater (*p* < 0.01) concentrations (reported as mg/100 g of sample) for total SFA, total MUFA, and total PUFA for geese supplemented I-Se and O-Se compared with geese fed CON. This was expected based on the differences in lipid composition observed in meat samples. Interestingly, geese supplemented O-Se had greater (*p* < 0.01) MUFA and less (*p* < 0.01) SFA compared with geese supplemented I-Se, while PUFA was not different (*p* > 0.05) between geese supplemented I-Se and O-Se. As a result, the ratio of PUFA:SFA was lower in geese supplemented I-Se compared with geese fed CON and geese supplemented O-Se.

While there is limited previous investigation on geese fatty acid profile, there are several previous research studies that have reported the fatty acid profile of meat from broiler chickens supplemented selenium. Overall, there are conflicting results regarding the impact of selenium supplementation on fatty acid profile of broiler chickens. Pappas et al. [51] reported no difference in SFA, PUFA, and PUFA:SFA of breast meat between broiler chickens supplemented 0.3 mg/kg of organic selenium (selenium-enriched yeast) and broiler chickens not supplemented with selenium; however, MUFA content of breast meat was reduced in broiler chickens supplemented 0.3 mg/kg of organic selenium compared with broiler chickens not supplemented with selenium. Kralik et al. [52] reported supplementing 0.3 mg/kg or 0.5 mg/kg of organic selenium (selenium-enriched yeast) to broiler chickens did not affect SFA concentrations in breast meat when compared with non-supplemented broiler chickens. Yet, broiler chickens supplemented with 0.3 mg/kg of organic selenium had less MUFA and greater PUFA compared with both non-supplemented broiler chickens and broiler chickens supplemented with 0.5 mg/kg of organic selenium [52]. Kralik et al. [53] reported supplementation with 0.5 mg/kg of organic selenium (selenium-enriched yeast) for broiler chickens lowered MUFA and increased n-3 PUFA in chicken thighs when compared with non-supplemented broiler chickens; however, no difference was observed for SFA or n-6 PUFA. del Puerto [54] reported no differences in SFA, MUFA, or PUFA for breast or thigh muscle for broiler chickens supplemented with 0.3 mg/kg of inorganic selenium (sodium selenite), broiler chickens supplemented with 0.3 mg/kg of organic selenium (selenomethionine), or broiler chickens not supplemented with selenium. Leskovec et al. [55] reported supplementing 0.2 mg/kg of selenium (source not provided) did not affect SFA, MUFA, or PUFA concentrations in the breast meat of chicken broilers.

### 3.4. pH

**Figure 1:**
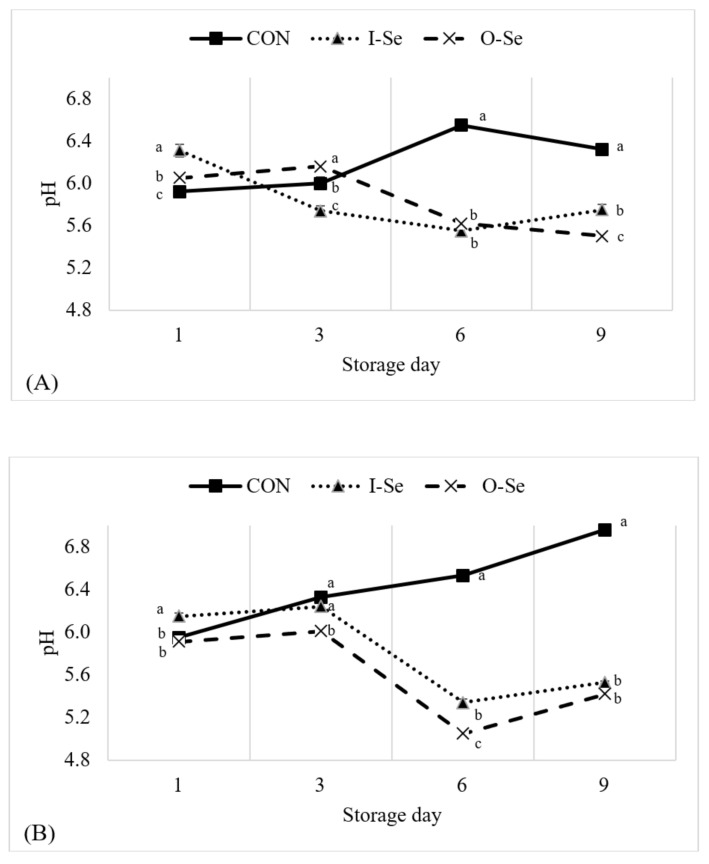
Effects of dietary supplementation with inorganic or organic selenium on the pH for goose meat (equal portions of the breast and thigh meat) (**A**) and liver (**B**) during 9 days of storage at 4 °C. Treatments were defined as CON: basal diet without supplemental selenium; I-Se: basal diet with 0.3 mg/kg inorganic selenium; O-Se: basal diet with 0.3 mg/kg organic selenium. Means within the same day with differing letters are different (*p* < 0.05).

pH of the meat samples over the storage period is presented in Figure 1A. There was a significant treatment effect (*p* < 0.05) for pH on each storage day. The most meaningful difference in pH of the meat samples was observed on day-6 and day-9 of the storage period where the pH of geese fed CON was greater than pH of geese supplemented I-Se and O-Se. pH of the liver over the storage period is presented in Figure 1B. Similar to the observations for meat samples, the most meaningful difference in pH of the liver was observed on day-6 and day-9 of the storage period where the pH of geese fed CON was greater than pH of geese supplemented I-Se and O-Se. It is well established that microbial growth during storage can affect pH [56,57], and data from the current study indicate that meat and liver samples from geese supplemented with selenium (both I-Se and O-Se) may behave differently in terms of their microbial stability.

While pH of muscle samples in selenium supplemented poultry has not been previously measured during an extended aerobic storage period as performed in this study, several previous studies have reported ultimate pH for muscle samples of broiler chickens at the time of fabrication (i.e., 24 to 48 h post-mortem). Perić et al. [58] reported no difference in breast muscle pH for broiler chickens supplemented with 0.3 mg/kg of inorganic selenium (sodium selenite) or 0.3 mg/kg of organic selenium (selenium-enriched yeast). Silva et al. [59] reported no differences in breast meat pH of broiler chickens supplemented with 0.3 to 0.5 mg/kg of inorganic (sodium selenite) or organic (selenomethionine) selenium. Leskovec et al. [55] reported no difference in breast muscle pH between broiler chickens fed selenium (source not provided) and broiler chickens not supplemented with additional selenium. Furthermore, Leskovec et al. [55] reported a consistent drop in pH from 24-h post-mortem to 48-h post-mortem for both broiler chickens fed selenium (5.92 to 5.84) and broiler chickens not supplemented with additional selenium (5.96 to 5.88). To our knowledge, no previous studies have investigated pH for liver samples from poultry supplemented with selenium. Greater research into the cause of the observed pH decline over time in both muscle and liver samples for selenium supplemented geese is warranted.

### 3.5. Total Phenolic Content

**Figure 2:**
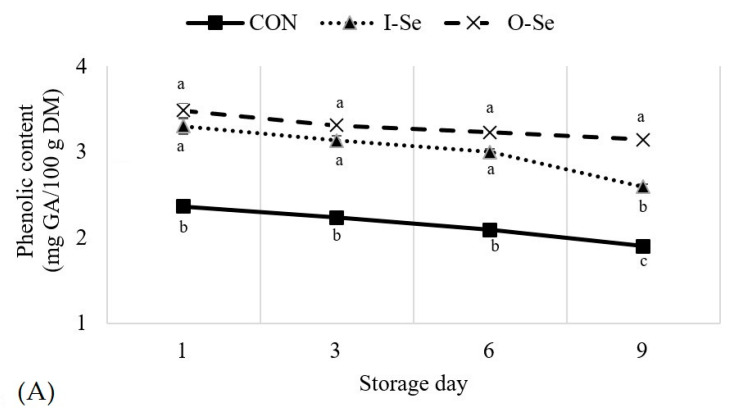

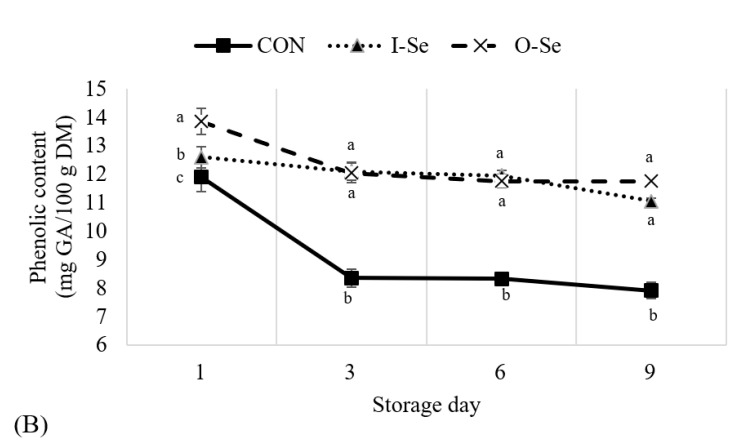
Effects of dietary supplementation with inorganic or organic selenium on the phenolic content for goose meat (equal portions of the breast and thigh meat) (**A**) and liver (**B**) during 9 days of storage at 4 °C. Treatments were defined as CON: basal diet without supplemental selenium; I-Se: basal diet with 0.3 mg/kg inorganic selenium; O-Se: basal diet with 0.3 mg/kg organic selenium. Means within the same day with differing letters are different (*p* < 0.05).

The change in phenolic content in meat samples and liver samples during the storage period is presented in Figure 2. Selenium supplementation increased (*p* < 0.05) the phenolic content of both meat samples and liver samples. The role of dietary selenium as an antioxidant has been well established [5,60,61]. Yet, research reporting on phenolic content of meat and liver samples from selenium supplemented poultry are actually inconclusive. Karadas et al. [62] reported selenium (selenium-enriched yeast) supplementation increased the accumulation of antioxidants in liver and plasma and improved the immune response in broiler chickens. Yet, Leskovec et al. [55] reported no difference in breast muscle α- and γ-tocopherol levels between broiler chickens fed selenium (source not provided) and broiler chickens not supplemented with additional selenium.

Surprisingly, the phenolic content in the meat samples and liver samples seemed to have been maintained over the storage period for both geese supplemented I-Se and O-Se. This was indicated by greater levels of phenolic content for the geese supplemented I-Se and O-Se at each storage day for the muscle samples and the liver samples. It is usually assumed that the level of phenolic compounds decreases during aerobic storage of fresh meat products [46], it was possible that greater selenium concentration in meat samples had an influence on phenolic content levels. A recent study by our research team [19] reported similar findings for the stability observed for phenolic content during prolonged periods of storage in tissue samples from geese supplemented with vitamin E. Furthermore, studies in other food products have indicated that greater selenium concentration led to greater stability of phenolic activity during storage, for example, selenium biofortification in apples [63] and in sweet basil [64] resulted in greater levels of phenolic content.

### 3.6. Lipid Oxidation

**Figure 3:**
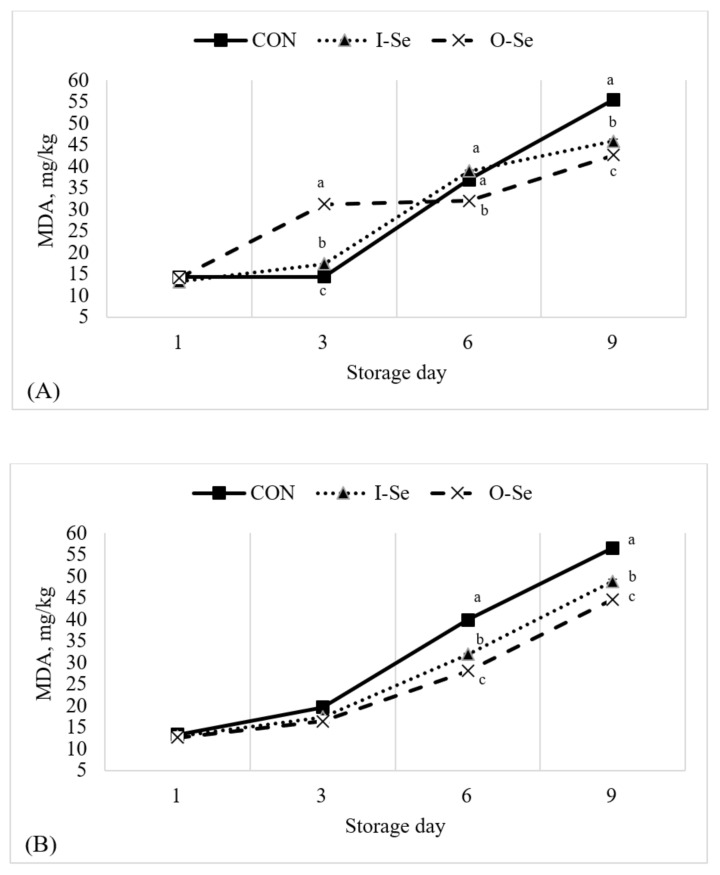
Effects of dietary supplementation with inorganic or organic selenium on the thiobarbituric acid reactive substances (TBARS) for goose meat (equal portions of the breast and thigh meat) (**A**) and liver (**B**) during 9 days of storage at 4 °C. Treatments were defined as CON: basal diet without supplemental selenium; I-Se: basal diet with 0.3 mg/kg inorganic selenium; O-Se: basal diet with 0.3 mg/kg organic selenium. Means within the same day with differing letters are different (*p* < 0.05).

The change in lipid oxidation, as measured using the TBARS assay, in meat samples and liver samples during the storage period is presented in Figure 3. The TBARS assay predicts lipid oxidation using a secondary oxidation product called malondialdehyde (MDA) where greater MDA levels indicate greater lipid oxidation. In meat samples, there was a significant treatment effect (*p* < 0.05) on day-3, day-6, and day-9 of the storage period. Generally, the results were inconsistent and difficult to summarize. MDA levels were greatest for meat samples from geese supplemented I-Se on day-3 and greatest for meat samples from geese fed CON on day-9 when compared with the other treatments. In liver samples, there was a significant treatment effect (*p* < 0.05) on day-6 and day-9 of the storage period. On each of these storage days, a consistent theme was apparent; liver samples from geese fed CON had the greatest MDA values and liver samples from geese supplemented O-Se had the lowest MDA values, while liver samples from geese supplemented I-Se were intermediate.

Overall, it can be concluded from this study that lipid oxidation, as measured using the TBARS assay, was generally unaffected when geese were supplemented with selenium. Many other studies have evaluated lipid oxidation of meat samples from broiler chickens supplemented with selenium and the results were unsurprisingly inconsistent. Ryu et al. [65] reported lower MDA levels in breast and thigh samples following 10 days and 12 days of refrigerated storage when broiler chickens were supplemented with varying concentrations of inorganic selenium (sodium selenite). Taulescu et al. [60] reported lower MDA levels following storage in meat samples sourced from broiler chickens fed flax seed and supplemented with vitamin E and selenium when compared with meat samples sourced from broiler chickens fed flax seed without supplementation of vitamin E and selenium. Kralik et al. [52] and Kralick et al. [53] reported lower MDA levels following storage in breast muscle and thigh muscle when broiler chickens were supplemented with 0.5 mg/kg of organic selenium (selenium-enriched yeast). Leskovec et al. [55] reported no difference in breast muscle MDA levels following fresh storage between broiler chickens fed selenium (source not provided) and broiler chickens not supplemented with additional selenium. Important considerations from each of these studies is the presence of other pro- and anti-oxidants in the diet (i.e., vitamin E) as selenium has been shown to have interactive roles with both dietary pro- and anti-oxidants [3]; as well as, dosage level of the supplemental selenium.

### 3.7. Total Volatile Basic Nitrogen

TVB-N is of interest during storage of meat products as it is considered an indication of freshness. Decomposition of protein results in accumulation of several volatile compounds including ammonia, dimethylamine, and trimethylamine. During storage, bacteria and proteolytic enzymes in poultry meat products can affect TVB-N values [19,39,66]. Cathepsin D is an endo-protease enzyme, which is distributed in the cell lysosome. The main function of cathepsin D is to degrade proteins and activate precursors of bioactive proteins in pre-lysosomal compartments [67]. Therefore, TVB-N content in poultry meat is an important chemical indicator to determine preservation of quality during storage [68].

**Figure 4:**
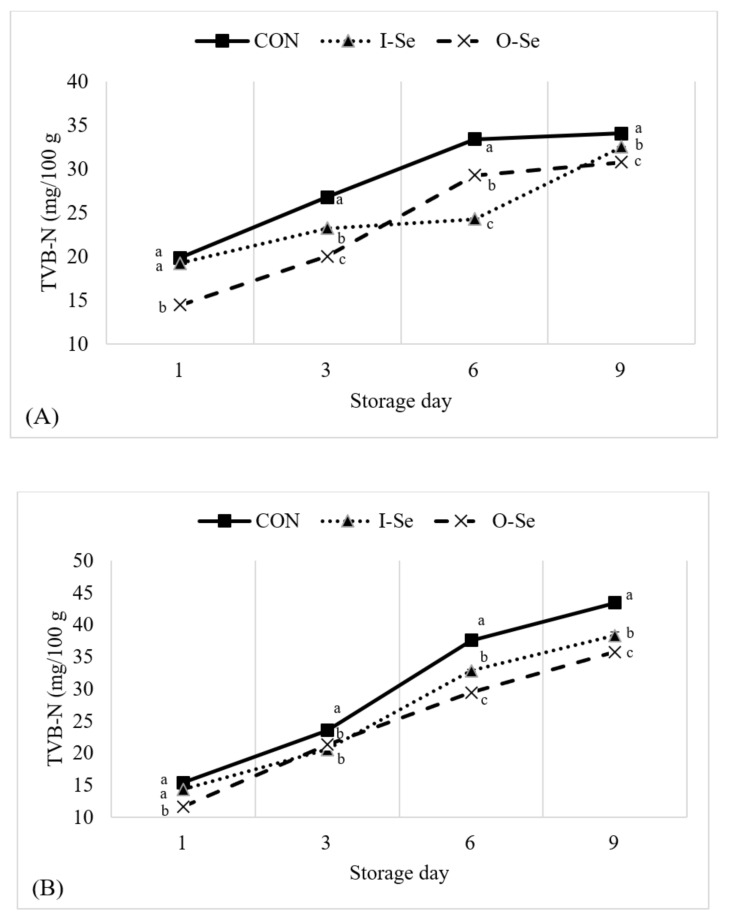
Effects of dietary supplementation with inorganic or organic selenium on the total volatile basic nitrogen (TVB-N) for goose meat (equal portions of the breast and thigh meat) (**A**) and liver (**B**) during 9 days of storage at 4 °C. Treatments were defined as CON: basal diet without supplemental selenium; I-Se: basal diet with 0.3 mg/kg inorganic selenium; O-Se: basal diet with 0.3 mg/kg organic selenium. Means within the same day with differing letters are different (*p* < 0.05).

The change in TVB-N value in meat samples and liver samples during the storage period is presented in Figure 4. Supplementation with either selenium diet (I-Se or O-Se) reduced (*p* < 0.05) the TVB-N content of both the goose meat samples and liver samples during the storage period. TVB-N increased during the storage period for both meat samples and liver samples. For meat samples, the most profound differences were observed on day-1, day-3, and day-6 of storage where geese supplemented I-Se and O-Se had lower TVB-N levels compared with geese fed CON. For liver samples, the magnitude of this difference was small and of questionable biological significance in most cases. At the conclusion of the storage period (on day-9)–TVB-N values of meat samples were 34.08 mg/100 g, 32.52 mg/100 kg, and 28.79 mg/100 g; and TVB-N values of liver samples were 43.45 mg/100 g, 38.34 mg/100 g, and 35.75 mg/100 g for the CON, I-Se, and O-Se treatments, respectively. From these observations, it can be concluded that the TVB-N values in both meat and liver samples were significantly lower for geese supplemented I-Se and O-Se compared with geese fed CON, yet the biological significance of such a minor magnitude of difference should be noted.

## 4. Conclusions

This study provided an in-depth assessment of meat and liver quality from geese supplemented with inorganic selenium (sodium selenite) and organic selenium (selenium-enriched yeast). Overall, it was concluded that supplementation of selenium in both the inorganic and organic form showed potential to improve goose meat and liver nutritional quality and shelf life. Specifically, meat and liver samples from geese supplemented I-Se or O-Se had greater lipid content compared with geese not supplemented with additional selenium, and both meat and liver from geese supplemented I-Se or O-Se had lower pH values, greater phenolic content, lower TBARS values, and lower TVB-N compared with geese not supplemented with additional selenium during and at the conclusion of a 9-day refrigerated storage period.

Future research endeavors to help compliment and expand upon these research initiatives should include a greater investigation of the mechanistic action of improved fat deposition associated with selenium supplementation of geese; as well as how the greater fat content in liver samples of selenium supplemented geese affects further processed liver products like *foie gras*.

## Figures and Tables

**Table 1:** Composition and nutrient levels of the basal diets.

Ingredients (%)	1 to 28 Day	29 to 56 Day
Corn	42.5	54.5
Wheat	24.0	26.0
Soybean meal	30.0	16.0
Dicalcium phosphate	1.0	1.0
Oyster shell	1.0	1.0
Edible salt (NaCl)	0.1	0.1
Mineral supplement ^1^	0.5	0.5
Vitamin supplement ^2^	0.5	0.5
L-Lysine	0.2	0.2
DL-methionine	0.2	0.2

^1^ Mineral supplement composition (per kg) consisted of the following: 24 g/kg manganese oxide, 16 g/kg iron sulfate, 3 g/kg copper sulfate, 0.2 g/kg calcium iodate, and 0.08 g/kg cobalt. ^2^ Vitamin supplement composition (per kg) consisted of the following: 36 × 10^7^ IU/kg vitamin A, 8 × 10^5^ IU/kg vitamin D_3_, 7 g/kg vitamin K_3_, 0.7 g/kg vitamin B_1_, 2.64 g/kg vitamin B_2_, 4 g/kg nicotinic acid, 12 g/kg pantothenic acid, 1.2 g/kg pyridoxine, 0.04 g/kg biotin, 0.4 g/kg folic acid, and 0.006 g/kg cobalamin.

**Table 2:** Effects of dietary supplementation with inorganic or organic selenium on the chemical composition for goose meat (equal portions of the breast and thigh meat) of native Azerbaijan geese.

		Treatments ^1^		
	Storage Day	CON	I-Se	O-Se
Moisture, %	day-1	68.64 ± 0.15 ^A,x^	68.38 ± 0.11 ^A,x^	66.56 ± 0.02 ^B,x^
	day-3	67.52 ± 0.21 ^A,xy^	65.82 ± 0.05 ^B,y^	65.45 ± 0.05 ^B,y^
	day-6	66.94 ± 0.15 ^A,y^	65.12 ± 0.010 ^B,y^	65.71 ± 0.06 ^B,y^
	day-9	64.44 ± 0.24 ^B,z^	64.67 ± 0.04 ^AB,z^	65.80 ± 0.03 ^A,y^
Protein, %	day-1	20.58 ± 0.17 ^B^	20.65 ± 0.04 ^B^	21.72 ± 0.06 ^A^
	day-3	20.58 ± 0.11 ^B^	20.67 ± 0.07 ^B^	21.85 ± 0.07 ^A^
	day-6	20.62 ± 0.16 ^B^	21.11 ± 0.08 ^AB^	22.12 ± 0.06 ^A^
	day-9	20.93 ± 0.17 ^B^	21.47 ± 0.06 ^AB^	22.12 ± 0.04 ^A^
Lipid, %	day-1	6.53 ± 0.34 ^C^	12.22 ± 0.001 ^A^	11.52 ± 0.012 ^B^
	day-3	6.28 ± 0.23 ^C^	12.24 ± 0.016 ^A^	11.44 ± 0.033 ^B^
	day-6	6.59 ± 0.24 ^C^	12.25 ± 0.029 ^A^	11.53 ± 0.005 ^B^
	day-9	6.95 ± 0.017 ^C^	12.42 ± 0.008 ^A^	11.54 ± 0.057 ^B^
Ash, %	day-1	1.86 ± 0.050 ^A^	1.37 ± 0.005 ^C^	1.73 ± 0.002 ^B^
	day-3	2.01 ± 0.130 ^A^	1.39 ± 0.001 ^B^	1.28 ± 0.007 ^C^
	day-6	2.12 ± 0.020 ^B^	1.47 ± 0.005 ^C^	2.34 ± 0.002 ^A^
	day-9	2.11 ± 0.160 ^A^	1.40 ± 0.001 ^B^	1.38 ± 0.004 ^B^

^A–C^ Means within the same row with differing upper-case letter superscripts are different (*p* < 0.05). ^x–z^ Means within column of the same parameter (treatment and day) with differing lower-case letter superscripts are different (*p* < 0.05). ^1^ Treatments were defined as CON: basal diet without supplemental selenium; I-Se: basal diet with 0.3 mg/kg inorganic selenium; O-Se: basal diet with 0.3 mg/kg organic selenium.

**Table 3:** Effects of dietary supplementation with inorganic or organic selenium on the chemical composition for liver of native Azerbaijan geese.

		Treatments ^1^		
	Storage Day	CON	I-Se	O-Se
Moisture, %	day-1	75.61 ± 0.05 ^A,x^	68.55 ± 0.07 ^C^	73.25 ± 0.05 ^B,x^
	day-3	75.03 ± 0.03 ^A,xy^	68.20 ± 0.22 ^C^	71.54 ± 0.05 ^B,y^
	day-6	74.51 ± 0.11 ^A,yz^	68.14 ± 0.04 ^C^	71.08 ± 0.03 ^B,z^
	day-9	74.46 ± 0.05 ^A,z^	68.13 ± 0.06 ^C^	71.95 ± 0.04 ^B,z^
Protein, %	day-1	13.38 ± 0.04 ^B^	13.64 ± 0.04 ^B^	15.77 ± 0.06 ^A^
	day-3	13.59 ± 0.09 ^B^	13.77 ± 0.01 ^B^	15.88 ± 0.05 ^A^
	day-6	13.94 ± 0.07 ^B^	13.88 ± 0.03 ^B^	16.13 ± 0.06 ^A^
	day-9	14.13 ± 0.09 ^B^	14.01 ± 0.02 ^B^	16.23 ± 0.02 ^A^
Lipid, %	day-1	6.26 ± 0.02 ^B^	8.23 ± 0.02 ^A^	8.30 ± 0.04 ^A^
	day-3	6.21 ± 0.03 ^B^	8.31 ± 0.01 ^A^	8.41 ± 0.02 ^A^
	day-6	6.25 ± 0.03 ^B^	8.32 ± 0.03 ^A^	8.42 ± 0.04 ^A^
	day-9	6.28 ± 0.01 ^B^	8.38 ± 0.02 ^A^	8.50 ± 0.01 ^A^
Ash, %	day-1	1.47 ± 0.003 ^A^	1.42 ± 0.002 ^B,x^	1.42 ± 0.001 ^B,x^
	day-3	1.41 ± 0.008 ^A^	1.10 ± 0.007 ^C,y^	1.20 ± 0.009 ^B,y^
	day-6	1.43 ± 0.008 ^A^	1.01 ± 0.004 ^C,yz^	1.18 ± 0.001 ^B,y^
	day-9	1.40 ± 0.002 ^A^	0.99 ± 0.005 ^C,z^	1.19 ± 0.002 ^B,y^

^A–C^ Means within the same row with differing upper-case letter superscripts are different (*p* < 0.05). ^x–z^ Means within column of the same parameter with differing lower-case letter superscripts are different (*p* < 0.05). ^1^ Treatments were defined as CON: basal diet without supplemental selenium; I-Se: basal diet with 0.3 mg/kg inorganic selenium; O-Se: basal diet with 0.3 mg/kg organic selenium.

**Table 4:** Effects of dietary supplementation with inorganic or organic selenium on the fatty acid profile for goose meat (equal portions of the breast and thigh meat) of native Azerbaijan geese.

	Treatments ^1^				
Fatty Acids, mg/100 g Sample	CON	I-Se	O-Se	SEM ^2^	*p*-Value ^3^
C9:0 (Pelargonic acid)	21.75 ^B^	111.20 ^A^	53.57 ^B^	14.87	0.01
C10:0 (Capric acid)	223.10 ^B^	961.10 ^A^	412.40 ^B^	102.15	0.01
C11:0 (Undecanoic acid)	18.78 ^B^	92.26 ^A^	37.44 ^B^	10.88	0.01
C12:0 (Lauric acid)	173.32 ^B^	317.11 ^A^	309.31 ^A^	35.62	0.05
C13:0 (Tridecylic acid)	21.75 ^B^	73.32 ^A^	31.10 ^B^	8.44	0.01
C14:0 (Myristic acid)	16.48 ^B^	60.49 ^A^	25.34 ^B^	8.12	0.02
C16:0 (Palmitic acid)	1122.94 ^B^	1963.14 ^A^	2024.06 ^A^	61.12	<0.01
C18:0 (Stearic acid)	972.00 ^B^	1412.60 ^A^	1424.40 ^A^	89.48	0.02
C20: 0 (Arachidic acid)	0.11	0.11	0.17	0.03	0.34
ΣSFA	2570.23 ^C^	4991.33 ^A^	4317.80 ^B^	58.33	<0.01
C14:1 (Myristoleic acid)	19.77	38.29	49.54	12.43	0.30
C16:1 (Palmitoleic acid)	113.68	138.7	187.78	21.37	0.12
C18:1 (Oleic acid)	1717.4 ^B^	2807.5 ^A^	3000.40 ^A^	79.16	<0.01
C20:1 (Gadoleic acid)	0.08 ^B^	0.11 ^AB^	0.19 ^A^	0.03	0.07
ΣMUFA	1850.93 ^C^	2984.57 ^B^	3237.91 ^A^	90.44	<0.01
C18:2 (Linoleic acid)	802.66 ^C^	1355.81 ^B^	1514.30 ^A^	26.57	<0.01
C18:3 (Linolenic acid)	1.33	1.21	1.54	0.16	0.21
C20:4 (Arachidonic acid)	677.10 ^B^	1104.70 ^A^	994.80 ^AB^	95.33	0.05
C22:6 (Docosahexaenic acid)	36.24 ^C^	84.32 ^B^	123.84 ^A^	7.04	<0.01
ΣPUFA	1517.33 ^B^	2546.04 ^A^	2634.47 ^A^	102.99	<0.01
PUFA:SFA	0.59 ^A^	0.51 ^B^	0.61 ^A^	0.02	0.04

^A–C^ Means within the same row with differing upper-case letter superscripts are different (*p* < 0.05). ^1^ Treatments were defined as CON: basal diet without supplemental selenium; I-Se: basal diet with 0.3 mg/kg inorganic selenium; O-Se: basal diet with 0.3 mg/kg organic selenium. ^2^ The maximum standard error of the mean (SEM) for each variable. ^3^ The *p*-value was reported for the main effect of dietary treatment.

## Data Availability

Data from this study will be made available upon request.
